# Cavitating and tigroid‐like leukoencephalopathy in a case of *NDUFA2*‐related disorder

**DOI:** 10.1002/jmd2.12094

**Published:** 2020-02-06

**Authors:** Marianna Alagia, Gerarda Cappuccio, Annalaura Torella, Alessandra D'Amico, Federica Mazio, Alfonso Romano, Simona Fecarotta, Giorgio Casari, Vincenzo Nigro, TUDP, Nicola Brunetti‐Pierri

**Affiliations:** ^1^ Department of Translational Medicine Federico II University Naples Italy; ^2^ Telethon Institute of Genetics and Medicine Naples Italy; ^3^ Medical Genetics, Department of Biochemistry, Biophysics and General Pathology University of Campania ‘Luigi Vanvitelli’ Naples Italy; ^4^ Department of Advanced Biomedical Sciences Federico II University Naples Italy; ^5^ Department of Neurosciences, Division of Neuroradiology Ospedale Santobono‐Pausilipon Naples Italy

**Keywords:** leukodystrophy, mitochondrial, *NDUFA2*

## Abstract

Biallelic variants in nuclear gene *NDUFA2* have been reported so far in only three children with variable presentations including Leigh syndrome or leukoencephalopathy. Herein, we report a further female child affected by *NDUFA2*‐related disorder presenting with cavitating and tigroid‐like pattern of leukodystrophy and without systemic biochemical abnormalities of mitochondrial disorders.

**SYNOPSIS**

Cavitating and tigroid leukoencephalopathy are features of *NDUFA2*‐related disorder.

## 1 INTRODUCTION

Leukodystrophies are a group of genetically heterogeneous disorders with variable clinical features and presentations. They typically present with motor disturbances, delayed development of motor skills, failure to acquire new skills or regression.^1^ Brain magnetic resonance imaging (MRI) with spectroscopy, tissue biopsies, enzyme assays, and DNA analyses are needed to achieve a definitive diagnosis.^2^ Although the development of new technologies, particularly exome sequencing (ES), has provided a major aid for diagnosis of leukodystrophies, recognition of specific MRI patterns remains highly valuable.

Leukodystrophy can be a feature of mitochondrial disorders such as complex I deficiency, the most common single mitochondrial enzyme deficiency.^3^ Complex I consists of 45 subunits, and 38 of them are encoded by nuclear DNA. Among these, *NDUFA2* encodes the NADH‐ubiquinone oxidoreductase subunit A2, a complex I accessory subunit, located in the distal matrix arm. Biallelic mutations in *NDUFA2* have been described in three patients exhibiting an early‐onset progressive neurodegenerative disorder.^4^, ^5^, ^6^ We here report *NDUFA2*‐related disorder in a further female patient presenting with cavitating and tigroid‐like leukoencephalopathy.

## 2 CASE REPORT

The child was born to first cousin parents of Asian descent after 40 weeks of uncomplicated gestation. Perinatal events were unremarkable. Her birth weight was 2.3 kg (<5th centile, *z*‐score = −2.8), length was 49 cm (29th centile) and head circumference was 36 cm (75th centile). She said her first words at 10 months of age and by the age of 18 months she was able to say 10 words. She was able to walk independently at 17 months. By 20 months of age, she experienced a slow regression of both motor and verbal skills without any apparent trigger. Over the next months, she regained limited mobility and few words. At 3 years of age, she had normal weight and height, but her head circumference was 45 cm (<5th centile, *z*‐score = −2.5). She had drooling, axial hypotonia but lower limbs hypertonia, with brisk reflexes and spastic equinovarus deformity of the feet. No dysmorphic features were noted. At the last evaluation at the age of 4 years, she developed generalized seizure with right centrotemporal spikes on EEG and she was started on levetiracetam.

**Figure 1:**
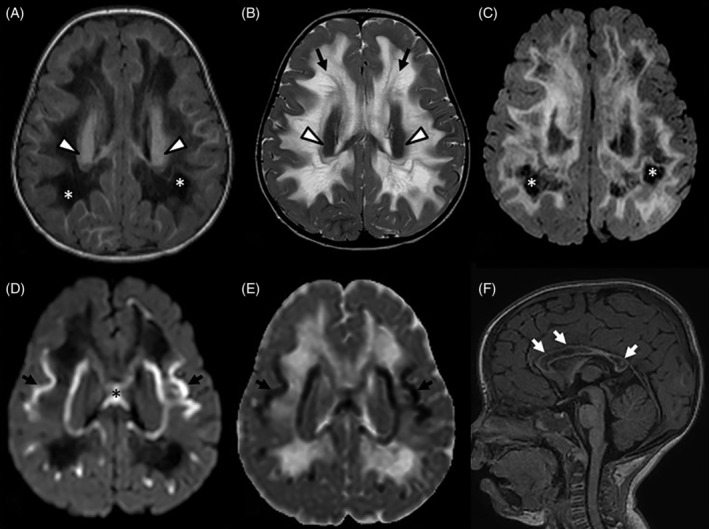
Axial spin‐echo T1‐ (A) and Turbo Spin‐Echo T2‐weighted sequences (B) show bilateral and confluent involvement of deep and peripheral supratentorial white matter. Note bilateral vacuolating pattern with cerebrospinal fluid‐like signal intensity on T1‐weighted and FLAIR sequences (asterisks in A and C) and the tigroid‐like pattern of the frontal deep white matter on T2‐weighted sequence (black arrows in B). The deep white matter of the corona radiata is spared bilaterally (white arrowheads in A and B). Diffusion weighted imaging (DWI; *b* = 1000) and apparent diffusion coefficient map show bilateral rims of restricted signal in the deep and juxtacortical white mater (black arrows in D and E) involving the trunk of the corpus callosum (black asterisk in D). The corpus callosum is diffusely vacuolated, with partial sparing of part of the isthmus (white arrows in F)

**Figure 2:**
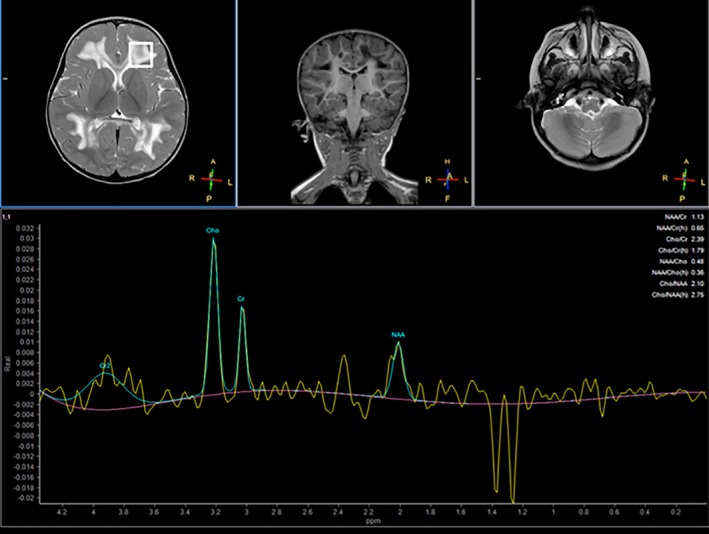
Single voxel ^1^H MR spectroscopy (PRESS TE 144) positioned in the left frontal white matter shows a prominent inverted double peak of Lactate between 1.2 and 1.4 ppm (ppm). Decreased *N*‐acetyl‐aspartate peak and increased Cho peak suggests neuronal damage

Brain MRI at 23 months of age showed diffuse and symmetric T1‐hypointensity and T2‐hyperintensity of periventricular and deep supratentorial white matter, sparing the deepest portions of the corona radiata (Figure 1A,B) and subcortical U‐fibers, especially at the posterior frontal convexities (Figure 1C). A tigroid‐like pattern of periventricular lesions was observed on T2‐weighted images, especially in the frontal lobes (Figure 1B). Bilateral cavitations in the deep and peripheral white matter of frontal, occipito‐parietal and posterior temporal regions were evident on T1‐weighted and FLAIR images (Figure 1A,C). Diffusion‐weighted imaging (DWI) showed bilateral rims with restricted diffusion in both deep and juxtacortical white matter, involving also the trunk of corpus callosum (Figure 1D,E), likely related to cytotoxic edema or intramyelinic edema. The corpus callosum was diffusely affected with a vacuolating pattern, partially sparing the isthmus (Figure 1F). Moreover, mild hyperintensity on T2‐weighted images was present in the olivary regions of the medulla and in pontine tegmentum. The signal intensity of basal ganglia and posterior internal capsules was normal. Magnetic resonance spectroscopic imaging (MRSI) by a single voxel technique using long TE of 288 to evaluate white matter showed increased choline/*N*‐acetyl‐aspartate ratio and a large double peak of lactate (Figure 2).

Blood levels of lactate and ammonium were within normal ranges, and no significant abnormalities were detected by plasma amino acids, acylcarnitines and very long chain fatty acids, serum transferrin isoelectric focusing, and urine organic acids. Array‐CGH analysis showed 3p14.1 (65766190_65819214 according to hg19) and 7q21.11 (85926414_85995934 according to hg19) microdeletions both present in controls and interpreted as nonpathogenic. The child was then enrolled in the Telethon Undiagnosed Diseases Program (TUDP) and following informed consent, her genomic DNA underwent ES that revealed a homozygous variant in *NDUFA2*: NM_002488: exon 2: c.170A>C:p.Glu57Ala (g.chr5:140026879T>G) that was confirmed by Sanger sequencing. Each parent was found to be heterozygous for the variant. The variant that was absent in ExAC and gnomAD, was predicted to be pathogenic by SIFT and polyphen with a CADD score of 31. Skin or muscle biopsies were not performed to evaluate the electron transport chain.

## 3 DISCUSSION

The diagnostic rate of ES in patients with white matter disorders ranges between 30 and 50%^6^, ^7^ with an increasing number of novel genetic conditions characterized. We describe a further patient with *NDUFA2*‐related disorder presenting with cavitating and tigroid‐like pattern of leukodystrophy. Prior to this report, biallelic variants in *NDUFA2* were described in only three children, presenting with Leigh syndrome or cystic leukodystrophy.^4^, ^5^ The clinical, biochemical and genetic findings of these three cases are summarized in Table 1 and compared with the case herein presented. All cases showed developmental delay and regression, although in at least two cases the loss of developmental skills was followed by either stabilization or regaining of some skills. In contrast to the other cases, the case reported by Hoefs et al appeared to have a more severe Leigh‐like presentation with hypertrophic cardiomyopathy and premature death.^4^ In three out of the four cases, evidence for increased lactate was detected by measurements of lactate in blood or in brain by MRSI.

**Table 1:** Comparison of clinical features among reported cases with *NDUFA2* variants

	Hoefs et al^4^	Perrier et al^5^		Present case
	Case 1	Case 2	Case 3^a^	Case 4
Gender	Male	Female	Female	Female
Age at last evaluation	11 months	12 years	4 years	4 years
Ethnicity	Turkish	Pakistani	Asian‐Indian	Asian
Consanguineity	+	+	−	+
Failure to thrive	N.A.	N.A.	+	+
Microcephaly	N.A.	N.A.	+	+
Developmental delay	+	+	+	+
Regression	+	+	+	+
Movement disorder	−	+ (spasticity and dystonia)	+ (no purposeful hand movements and upper motor neuron signs)	−
Eye abnormalities	+ (optic nerve atrophy)	−	−	+ (altered visual potentials)
Hearing loss	N.A.	N.A.	N.A.	+
Epilepsy	+	.+	−	+
Cardiac involvement	+ (hypertrophic cardiomyopathy)	−	−	−
Clinical progression	Death at 11 months	Episodes of hypoketotic hypoglycemia and hyperammonemia (likely related to systemic carnitine deficiency); developmental regression until age 12 months followed by stabilization	Able to walk with walker for short distance. Progression of brain MRI changes	Regression followed by regaining of motor and language skills
Lactic acidosis	+	−	−	−
ETC	Complex I deficiency^b^^,^ c	Complex I deficiency^c^	N.A.	N.A.
Brain MRI findings	Cerebral atrophy, corpus callosum hypoplasia, demyelinization of cortico‐spinal tract; subacute necrotizing encephalomyelopathy^d^	White matter changes with cysts of periventricular and subcortical regions, posterior limb of internal capsule	White matter changes with cysts of periventricular and subcortical regions, posterior limb of internal capsule, middle cerebellar peduncle	White matter changes with cysts of periventricular and supratentorial white matter; tigroid‐like lesions
Brain MRSI	N.A.	N.A.	Large lipid/lactate peak; low NAA peak	Lactate peak; increased choline/NAA ratio
Co‐morbid conditions	–	Primary systemic carnitine deficiency due to homozygous *SLC22A5* mutation	−	−
*NDUFA2* variantsNucleotide change	Homozygous c.208+5G>A	Homozygous c.134A>C	Compound heterozygous *Allele 1*: c.134A>C *Allele 2*: c.225del	Homozygous c.170A>C
Protein change	Impaired splicing of exon 2; *NDUFA2* mRNA not detected	p.Lys45Thr	*Allele 1*: p.Lys45Thr *Allele 2*: p.Asn76Metfs*4	p.Glu57Ala

Abbreviations: ETC, electron transport chain; MRI, magnetic resonance imaging; NAA, *N*‐acetyl‐aspartate; N.A., not available.

a Case also reported by Vanderver et al^6^ (1) as LD_0821.

b Muscle tissue.

c Skin fibroblasts.

d Images of the MRI were not included.

Although MRI images were not available, the pattern of neuroimaging abnormalities of the first reported patient with *NDUFA2* defect appears to be different compared to the present case and the two other cases reported by Perrier et al.^4^, ^5^ This first case with *NDUFA2* defect indeed showed an early‐onset of demyelination of cortico‐spinal tracts and subacute necrotizing encephalomyelopathy, as observed in Leigh syndrome.^4^ Consistent with the MRI findings of our case, the two children reported by Perrier et al showed confluent T2‐hyperintense and cystic changes of supratentorial white matter without involvement of basal ganglia.^5^ In contrast to the cases by Perrier et al,^5^ our case had an involvement of the corpus callosum, a tigroid‐like pattern of the deep white matter, and restriction of the DWI‐signal.

A tigroid‐like pattern of white matter lesions has been typically described in Pelizaeus‐Merzbacher disease and lysosomal storage disorders, particularly metachromatic leukodystrophy and globoid cell leukodystrophy.^8^ Only one case with Kearns‐Sayre syndrome, a mitochondrial disease, has been previously found to have radial stripes.^9^ Pathology studies in lysosomal storage disorders suggested that the stripes correspond to spared myelin or perivenular clusters of globoid cells with lipid storage.^9^ Whether they represent perivenular spared myelin also in mitochondrial disorders needs to be established. Nevertheless, our case suggests that *NDUFA2*‐related disorder can present with both cavitating and tigroid‐like pattern leukoencephalopathy on neuroimaging.

## CONFLICT OF INTEREST

The authors report no disclosures relevant to the manuscript.

## AUTHOR CONTRIBUTIONS

Marianna Alagia, Gerarda Cappuccio and Nicola Brunetti‐Pierri: intellectual content and original manuscript draft. Annalaura Torella, Alessandra D'Amico, Federica Mazio, Alfonso Romano, Simona Fecarotta, Giorgio Casari, and Vincenzo Nigro: intellectual content.
